# Supplementary material for: A Lightweight and Low-Voltage-Operating Linear Actuator Based on the Electroactive Polymer Polypyrrole
Source: Polymers (Basel). 2023 Aug 18;15(16):3455. doi: 10.3390/polym15163455 (PMC10459993; doi:10.3390/polym15163455)
Supplement: Supplementary file 1 [file polymers-15-03455-s001.zip › polymers-2523114-supplementary.pdf]

Supplementary Information

A Lightweight and Low-voltage-operating Linear Actuator Based on the Electroactive Polymer Polypyrrole

Yeji Kim[1]*, Yasukazu Yoshida[2]

[1] Advanced Technology Research Dep., LG Japan Lab Inc., LG Yokohama Innovation Center 7F, 1-2-13, Takashima, Nishi-ku, Yokohama-shi, Kanagawa 220-0011, Japan; yeji2.kim@lgjlab.com

[2] yasukazu.yoshida@lgjlab.com

* Correspondence: yeji2.kim@lgjlab.com; Tel.: Tel: +81-3-6703-8506, Fax: +81-3-6703-8515

To create an atmospheric-operable linear actuator, we attempted to fabricate a tube-like shaped PPy film that can operate in a limited space. The polymerization conditions are the same as those used for the preparation of PPy films in the main text. The electrochemical polymerization temperature was kept at 10°C, and a Ni wire with a diameter of 1.2 mm and a length of 12 cm was used as the working electrode. The polymerization solution consisted of a mixture of PE and DEP solvents with TEATFSI and TBABF4 as electrolytes, and it also contained 0.15M Pyrrole. The current density was set at 0.1 mA/cm^2, and the polymerization was conducted for 20 hours. After the polymerization was completed, the tube-shaped PPy was swollen with acetone as described above, and then removed from the Ni wire. To preserve its shape, the removed PPy tube was dried by transferring it into a plastic rod (Figure S1). As shown in Figure S1, the PPy film was removed from the plastic rod after drying and cut to the required length for further use.

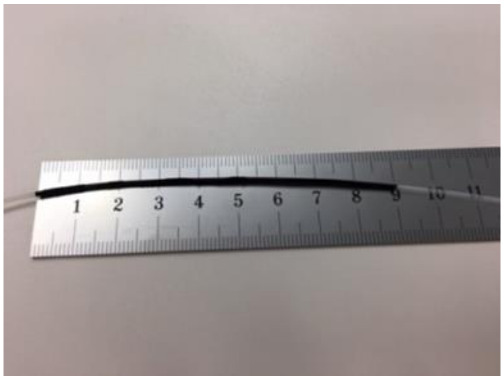

Figure S1. The photograph of the PPy film electrochemically polymerized on a Ni wire using polymerization solution III-2 for an atmospheric-operable linear actuator, taken during the drying process.

Table S1 presents the electrical conductivity of PPy films electrochemically polymerized under 6 different polymerization solutions, as shown in Table 1 of this paper. The electrochemical polymerization of PPy film was conducted using the same method as described in section 2.2. The electric conductivity of PPy films was measured using a standard 4-probe technique with a Loresta-GP (Mitsubishi Chemical). When comparing the conductivity of the PPy films polymerized using $TBABF_4$ and TEATFSI, $TBABF_4$ exhibited significantly higher conductivity. These results are believed to be attributed to the differences in electrolytes, causing changes in the morphology of the polymerized polymers.

Table S1. The electrical conductivity of the electrochemically polymerized films using six different polymerization solutions, each formed by combining three types of electrolytes with two types of solvents.

	Conductivity [S/cm]
Ⅰ-1	198
Ⅰ-2	138
Ⅱ-1	63
Ⅱ-2	71
Ⅲ-1	84
Ⅲ-2	147

Figure S2 shows actual photographs of the PPy films electrochemically polymerized using three types of electrolytes. As shown in Figure S1 (a), the film using $TBABF_4$ exhibited a smooth and dense surface structure, and no significant shape changes were observed even after polymerization and drying. On the other hand, films using (b) TEATFSI only and (c) TEATFSI with TBABF4 exhibited a sponge-like and very bulky structure, and significant shrinkage was observed during drying.

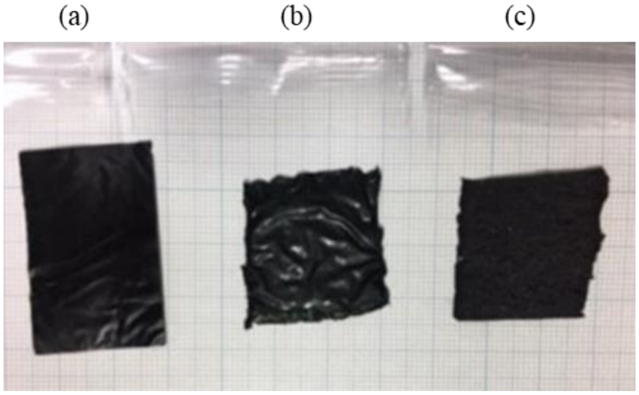

Figure S2. The photographs of the PPy films electrochemically polymerized under polymerization solution containing electrolyte (a) TBABF4, (b) TEATFSI, and (c) mixed electrolyte with TEATFSI and TBABF4.

Figure S3 presents SEM images of PPy films under the same conditions as the three polymerization solutions mentioned in Figure S2 with (a) showing a smooth surface, while (b) and (c) demonstrating a very bulky structure.

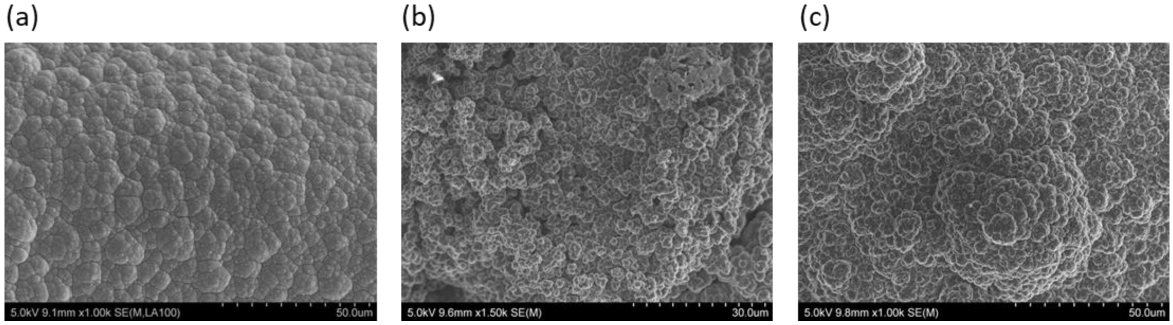

Figure S3. SEM images of PPy film polymerized under polymerization solution, Ⅲ-2 in Table 1

The bulky structure is highly beneficial for actuator stretchability; however, it also affects the film's durability and conductivity, resulting in a trade-off relationship. T To determine the most suitable conditions for practical applications, it is essential to consider various aspects such as the actuator's stretchability, generating force, conductivity, and film morphology, and strength. Utilizing the obtained results as feedback will help guide the optimal conditions for further development and application.
